# Hospital-induced immobility – a backstage story of lack of chairs, time, and assistance

**DOI:** 10.1186/s12877-024-05286-6

**Published:** 2024-08-24

**Authors:** Katrine Storm Piper, Martin Oxfeldt, Mette Merete Pedersen, Jan Christensen

**Affiliations:** 1 Department of Occupational Therapy and Physiotherapy, Copenhagen University Hospital, Rigshospitalet, Valdemar Hansens Vej 1-23, Glostrup, 2600 Denmark; 2 Department of Clinical Research, Copenhagen University Hospital, Hvidovre, Denmark; 3 Department of Clinical Medicine, University of Copenhagen, Copenhagen, Denmark; 4 Department of Public Health, University of Copenhagen, Copenhagen, Denmark

**Keywords:** Mobilisation, Rehabilitation, Older adults, Inactivity, Hospitalisation

## Abstract

**Background:**

Inactivity and bedrest during hospitalisation have numerous adverse consequences, and it is especially important that older patients are mobile during hospitalisation. This study aimed to identify whether the introduction of formal education of clinical staff and a Mobilisation Initiative (MI) could increase mobilisation of patients in a geriatric and a medical ward. Furthermore, to explore patients’ and health care staffs’ view on facilitators and barriers for mobilisation during hospitalisation.

**Methods:**

The study was a pragmatic clinical study. Both qualitative and quantitative methods were used. The patients’ level of mobilisation was obtained through short interview-based surveys and observations. Focus group interviews and formal education of clinical staff was initiated to increase awareness of mobilisation along with the implementation of a MI.

**Results:**

596 patient surveys were included. Of all patients, 50% in the geriatric ward and 70% in the medical ward were able to independently mobilise. The highest percentage of patients sitting in a chair for breakfast and lunch in the geriatric ward was 57% and 65%, and in the medical ward 23% and 26%, respectively. A facilitator for mobilisation was interdisciplinary collaboration, and barriers were lack of chairs and time, and the patients’ lack of help transferring.

**Conclusions:**

This study adds new knowledge regarding the lack of in-hospital mobilisation in geriatric and medical departments. Mealtimes are obvious mobilisation opportunities, but most patients consume their meals in bed. A potential for a MI is present, however, it must be interdisciplinarily and organisationally anchored for further investigation of effectiveness.

**Trial registration:**

Retrospectively registered at ClinicalTrials.gov with the trial number NCT05926908.

**Supplementary Information:**

The online version contains supplementary material available at 10.1186/s12877-024-05286-6.

## Background

In older adults, inactivity and bedrest during hospitalisation can lead to numerous adverse consequences, including loss of muscle mass and strength, loss of functional independence, and risk of re-hospitalisation and death [1–5]. Moreover, older adults have an impaired ability to recover compared with younger adults [6, 7], and those who are discharged with new disabilities have poor long-term recovery [8, 9]. The risk of new disabilities and loss of functional independence is a significant concern for older adults for whom maintenance of functional independence is a highly prioritised health outcome [10].

New disabilities and loss of function during hospitalisation are commonly known as hospital-associated disabilities (HADs) [5, 11]. According to a meta-analysis, the prevalence of HADs is 30% (range 17–61%) in acutely hospitalised older adults, and this number has remained stable in the last three decades despite shorter lengths of stay [11, 12]. Multiple risk factors for the development of HADs have been identified and are, to some extent, considered iatrogenic and preventable [13, 14]. One of these factors is low in-hospital activity, which has recently been linked with HADs in acutely admitted older adults [15].

Studies have shown that older patients are inactive for a large amount of the day during hospitalisation, independent of their functional ability on admission [5, 16–20]. Previously, we showed that older patients acutely admitted for medical illness spent 17 h lying, 5.1 h sitting, and only 1.1 h standing or walking per day [16], and took a median of 728 steps per day during hospitalisation [21]. This is far less than the recommended amount of activity of 7,000–10,000 steps per day for healthy older adults and 4,600-6,500 steps per day for older adults with chronic disabilities [22]. Similarly, Brown et al. investigated levels of in-hospital mobility (e.g. lying, sitting and walking) and reported that 48% of acutely admitted older patients had low or moderate levels of mobility throughout their hospital stay, equivalent to being mobilised to walk only twice per day on average [5]. Similar findings are synthesised in a systematic review showing that 93–98.8% of acutely admitted medical or surgical adult patients spent their in-hospital time in bed, and most of them walked less than 1000 steps per day [17]. Moreover, we recently showed that the amount of activity doubles immediately after discharge [21], indicating that older adults have the potential to be more physically active during hospitalisation.

Although low in-hospital activity is known to be associated with a range of poor outcomes, to date, there is no gold standard for how to ensure physical activity during hospitalisation in older adults [23], and a range of barriers to in-hospital physical activity has been put forward in a recent scoping review [24]. Among others, a lack of in-hospital mobility can occur due to a hospital culture in which bedrest is a natural part of being hospitalised and where no profession takes on the responsibility of ensuring mobilisation [25]. In addition, the importance of clear professional roles and responsibilities regarding older adults’ physical activity during hospitalisation, as well as organisational factors supporting physical activity, has been underlined in newly published international recommendations for older adults’ physical activity during hospitalisation [23]. Therefore, the aim of this pragmatic clinical study was to identify whether the introduction of formal education of clinical staff and a Mobilisation Initiative could increase the number of patients mobilised for breakfast and lunch among patients admitted to geriatric and medical wards.

## Methods

### Study design

This study employed an exploratory longitudinal design and was conducted as a pragmatic clinical study. Both qualitative and quantitative methods were used to obtain a picture of in-hospital mobility.

### Setting

This study was conducted between April 2021 and December 2021 at Copenhagen University Hospital, Rigshospitalet, and participants were included from two medical wards: a geriatric ward that specialises in treating older patients, mainly ≥ 65 years, with acute illness, chronic diseases, and physical and/or cognitive problems, and a medical ward that treats adult patients ≥ 18 years (primarily older patients) with cardiac, respiratory, endocrinological, and nephrological diseases. Each ward consisted of 20 beds placed in single-, double-, or triple-bed hospital rooms, with the possibility of placing two to three extra beds in the corridors during peak periods. A nursing staff group, an occupational therapist, and two to three physiotherapists were connected to each ward. In the geriatric ward, all patients were entitled to physiotherapy as part of the in-hospital treatment. In the medical ward, patients only received physiotherapy when they had a referral from the doctor. In both wards, according to local guidelines, all health care professionals should focus on mobilising patients as much as possible throughout hospitalisation. Usual practice was for the nursing staff to mobilise the patients to a chair for breakfast and lunch. Due to the Covid-19 pandemic, access to a separate dayroom with dining tables was prohibited in both wards.

### Participants

All patients in the geriatric and medical wards at Copenhagen University Hospital, Rigshospitalet, were considered eligible for participation and were consecutively screened for inclusion by the primary investigator (PI) (KSP). Patients were excluded if the ward physicians noted in the electronic medical records that they: (1) were moribund or delirious (o*ut of respect for the patients who were considered to prefer to not be disturbed with research questions)*,* or (2)* were isolated, as this would expose project staff and thereby other patients to unnecessary risk of infection during the Covid-19 pandemic. Also, patients who required an interpreter were excluded since the availability of interpreters was limited.

### Procedures and data sources

#### Self-reported level of patient mobilisation and observations of patients and the environment

**Fig. 1:**
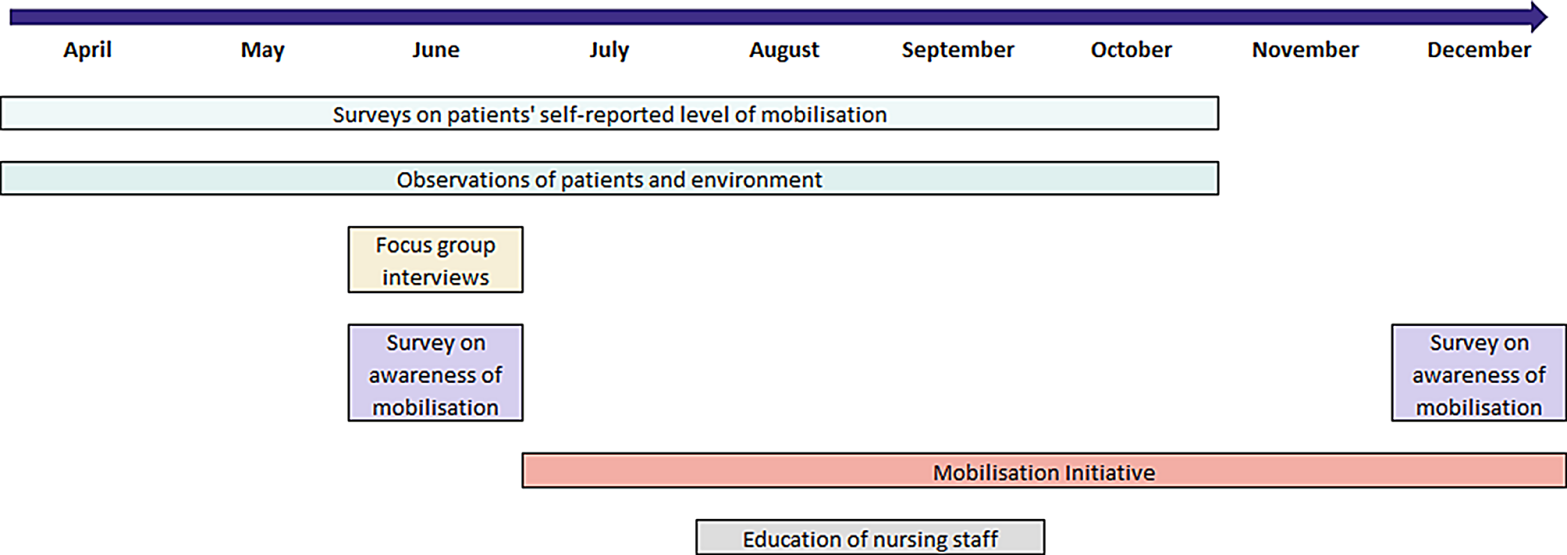
Timeline mobilisation project

Baseline assessments were conducted in April 2021, and the assessments were repeated every month during the study period (Fig. 1). Two days at the beginning of each month, surveys of the patients’ self-reported level of mealtime mobilisation and observations of the patients’ mobilisation level and environment at mealtimes were conducted (Fig. 1). Surveys and observations were conducted for all patients present in the wards. Thus, patients who were hospitalised on both survey days each month were observed/interviewed both days.

##### Self-reported level of patient mobilisation

After breakfast and lunch, KSP obtained data regarding each patient’s self-reported mealtime mobilisation level through interview-based surveys, including structured follow-up questions (Appendix 1). All surveys with the patients were conducted by KSP, and the selection of days depended on the possibility of her presence in the wards. The patients were asked, “How do you get from the bed to a chair?” to identify the patient’s self-reported level of mobilisation, from lying or sitting in bed to sitting in a chair near the bed at the time of the question. If in doubt, the patients were asked to report how they managed the latest transfer from the bed to a chair near the bed. The patients were asked to answer the question on a 5-item ordinal scale: independently, with supervision, with the physical help of one person, with the physical help of two persons, or never sit in a chair. Additionally, the patients were asked, “Where did you consume your breakfast/lunch?” referring to their latest meal. The patients answered on a 5-item categorical scale: lying in bed, sitting in bed, sitting on the edge of the bed, sitting in a chair, or did not have breakfast/lunch. If the patients answered that they did not sit in a chair, they were asked, “What was the reason you did not sit in a chair for breakfast/lunch?”

##### Observation of patients and Environment

During breakfast and lunch, the nursing staff, physiotherapists at the ward, and KSP observed the mobilisation status of all patients in the wards using an observation checklist (Appendix 2). Each patient’s observed mobilisation status was based on how breakfast and lunch were consumed and categorised as lying in bed, sitting in bed, sitting on the edge of the bed, or sitting in a chair. Furthermore, the presence and placement of chairs for each patient were registered using observation checklists.

### Focus group interviews

In June 2021, focus group interviews were conducted with health care professionals at the wards to explore the acceptability and demand for the contents of the Mobilisation Initiative (described below), including the identification of facilitators and barriers to mobilisation (Fig. 1). A semi-structured interview guide for the focus group interviews was developed in advance by KSP and a physiotherapist (DBS) with clinical experience in focus group interviews (Appendix 3). The guide was based on inspiration from former studies on barriers and facilitators for the mobilisation of patients [25, 26]. The ward nurses identified staff who would be interested in participating and available on the days of the interviews. The interviews were planned on days with the availability of both nursing staff and therapists. At each ward, one interview was planned with three nursing staff (one nurse and two care assistants), a physiotherapist, and an occupational therapist who all volunteered to participate. On the day of the interviews, however, only one nursing staff member and one physiotherapist at the geriatric ward and two nursing staff members and one occupational therapist at the medical ward were able to attend the interviews. The interviews took place in a quiet room in a nearby ward. The interviews were moderated by DBS, who was not employed at either ward. First, DBS introduced herself and the purpose of the focus group interview, assured the participants of their anonymity, and emphasised the importance of all participants’ contributions. Then, all participants introduced themselves. Hereafter, DBS used a semi-structured interview guide that consisted of open-ended questions to facilitate discussions on facilitators and barriers to mobilisation of patients during hospitalisation at their ward and in general, and to allow for in-depth enquiry of the participants’ answers (Appendix 3) [27]. Elaboration probes were used by DBS to invite all participants into the discussion [27]. The focus group interviews were observed by KSP, who only contributed when clarification of specific questions was needed. Both focus group interviews were recorded with a smartphone using the recorder app TapeACall Pro (https://www.tapeacall.com/). The participants gave verbal consent to record the interviews.

#### Awareness of mobilisation survey

To assess the nursing staff’s awareness of and confidence in the mobilisation of the patients, a short survey on mobilisation awareness was distributed to the nursing staff at both wards in June (before initiation of the Mobilisation Initiative) and December (Fig. 1) (Appendix 4). The surveys were accessible for a month with regular reminders of completion. The awareness of mobilisation among the nursing staff was explored within the terms of their self-reported sense of confidence, competencies, and daily awareness of the patients’ mobilisation through the questions: “Do you feel confident mobilising your patients to sit in a chair at mealtimes?,” “To what degree do you feel prepared to do a safe transfer from the bed to a chair with a patient you do not know?,” and “How often do you talk to your colleagues about mobilisation?” The nursing staff were asked to answer the questions on 5-item Likert scales: never, less than half of the time, half of the time, more than half of the time, and every time; and to a very low degree, to a low degree, neither/nor, to a high degree, and to a very high degree. The nursing staff was also asked to report the years employed at the ward.

#### Mobilisation Initiative

A Mobilisation Initiative with the aim of enhancing mobilisation was outlined before baseline assessments of patient mobilisation status. The Mobilisation Initiative consisted of physiotherapists assisting nursing staff in mobilising patients to sit in a chair for breakfast and lunch. With this initiative, a physiotherapist rounded each ward in the morning and for lunch on weekdays, asking the nursing staff if any help was needed to mobilise the patients. The physiotherapist provided guidance and/or physical assistance to the nursing staff, mobilising the patients in cases of uncertainty of the patient’s mobilisation level or when more than one person was needed to mobilise a patient. This approach was, contrary to usual practice, a proactive initiative with the physiotherapists offering their assistance at mealtimes.

The Mobilisation Initiative was launched in July 2021 and was carried out throughout the remainder of the study period (Fig. 1). The utilisation of the Mobilisation Initiative was recorded as the daily number of patients referred to and mobilised in the Mobilisation Initiative at each ward through a checklist filled out by the physiotherapists conducting the Mobilisation Initiative. Likewise, it was recorded when the physiotherapists occasionally mobilised patients not referred from the nursing staff (e.g., when the patients only needed supervision or a verbal prompt to transfer from the bed to a chair). Serious and non-serious adverse events and possible unintended effects of the Mobilisation Initiative were registered.

Evaluations with physiotherapists and nursing staff were conducted at regular intervals by KSP to handle emerging challenges and maintain focus on the Initiative. Procedures evaluated as not working were discontinued, and new methods were introduced, such as the physiotherapists assisting in the placement of chairs when missing. Simultaneously, facilitators and barriers to the Mobilisation Initiative verbalised by the staff were recorded.

#### Formal education

Parallel to the Mobilisation Initiative, formal education, including information on the consequences of immobilisation, was organised for all nursing staff employed at the two wards with the purpose of enhancing confidence, competencies, and awareness of mobilisation (Fig. 1). The education was led by KSP and arranged according to the nursing staff’s possibility of attendance on two days, one in August and one in September. The education included time for discussion about mobilisation. The number of nursing staff attending formal education was registered. During the formal education sessions, statements on facilitators and barriers to mobilisation, including lack of confidence and competencies, were registered during the discussion on mobilisation among attendees.

#### Demographic data

Data on age and the number of patient hospitalisation days were collected through the electronic medical records of all patients.

### Data management

Data forms for all sources were developed, and data were continuously entered into the Research Electronic Data Capture System (REDCap). To prevent errors, data forms in REDCap were prepared with safety margins prior to entering data, e.g., age only accepted from 18 to 110 years. Furthermore, the data were cross-tabulation validated.

### Statistical methods

Data were descriptive and reported as the means and standard deviations (SD) for continuous normally distributed data and as the median and range for non-normally distributed continuous data. Categorical data for observed and self-reported levels of mobility were reported as frequencies and percentages (%). The presence and placement of chairs were dichotomised as either present or absent and either placed at the bedside or not, respectively. The number of patients referred to the Mobilisation Initiative, the number of those referred who were mobilised, and the number of patients identified and mobilised without referral by the Mobilisation Initiative were reported as average numbers (n) throughout the study period. Data on mobilisation were primarily based on interview-based surveys. As data from the observation checklists and from the interview-based surveys were 80–100% identical, missing data from the interview-based surveys were replaced with data from the observation checklists when possible. All data were collected monthly. Data analysis was conducted using IBM SPSS Statistics for Windows, version 25 (IBM Corp., Armonk, N.Y., USA).

Data from focus group interviews were analysed using qualitative content analysis [28]. Both interviews with the staff at the geriatric and medical wards were used as the unit of analysis. First, the interviews were transcribed verbatim and read in full length. Second, meaning units were identified and condensed. Subsequently, the condensed meaning units were abstracted and labelled with a code accordingly. The codes were compared and categorised to represent the manifest content. Finally, the latent content, and underlying meaning of the categories were identified and expressed into themes.

## Results

### Self-reported level of mobilisation and observations of patients and the environment

**Fig. 2:**
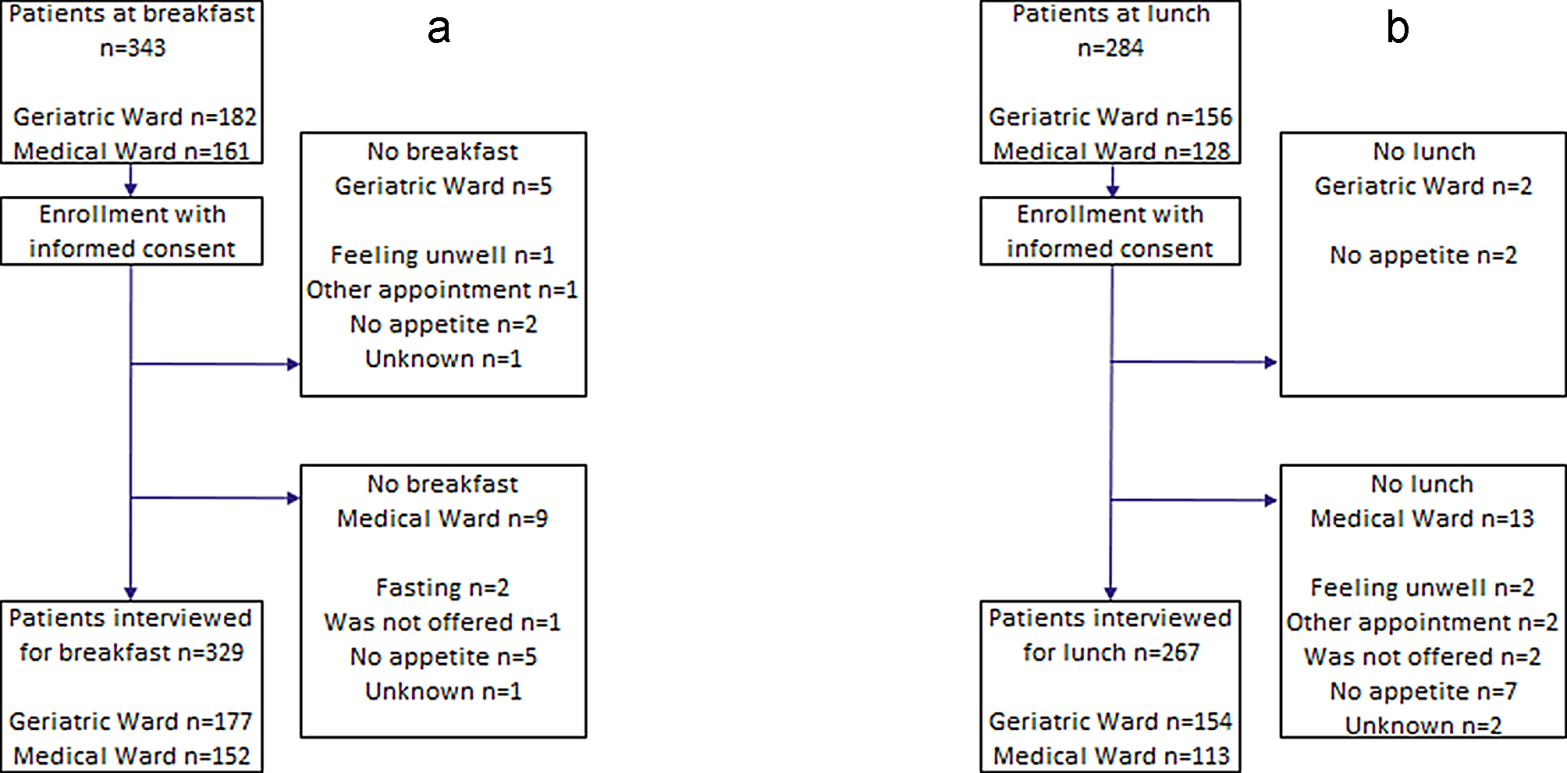
**a** Flowchart of patients’ self-reported level of mobilisation at breakfast. **b** Flowchart of patients’ self-reported level of mobilisation at lunch

A total of 596 patient surveys on self-reported levels of mobilisation were conducted from April to October (Fig. 2a and b). The mean age at the geriatric ward was 81.6 (SD 9.2) years, and at the medical ward, it was 72.1 (12.8) years. Self-reported levels of mobilisation were obtained after a median of three days of hospitalisation, with a range of 1–26 days.

**Fig. 3:**
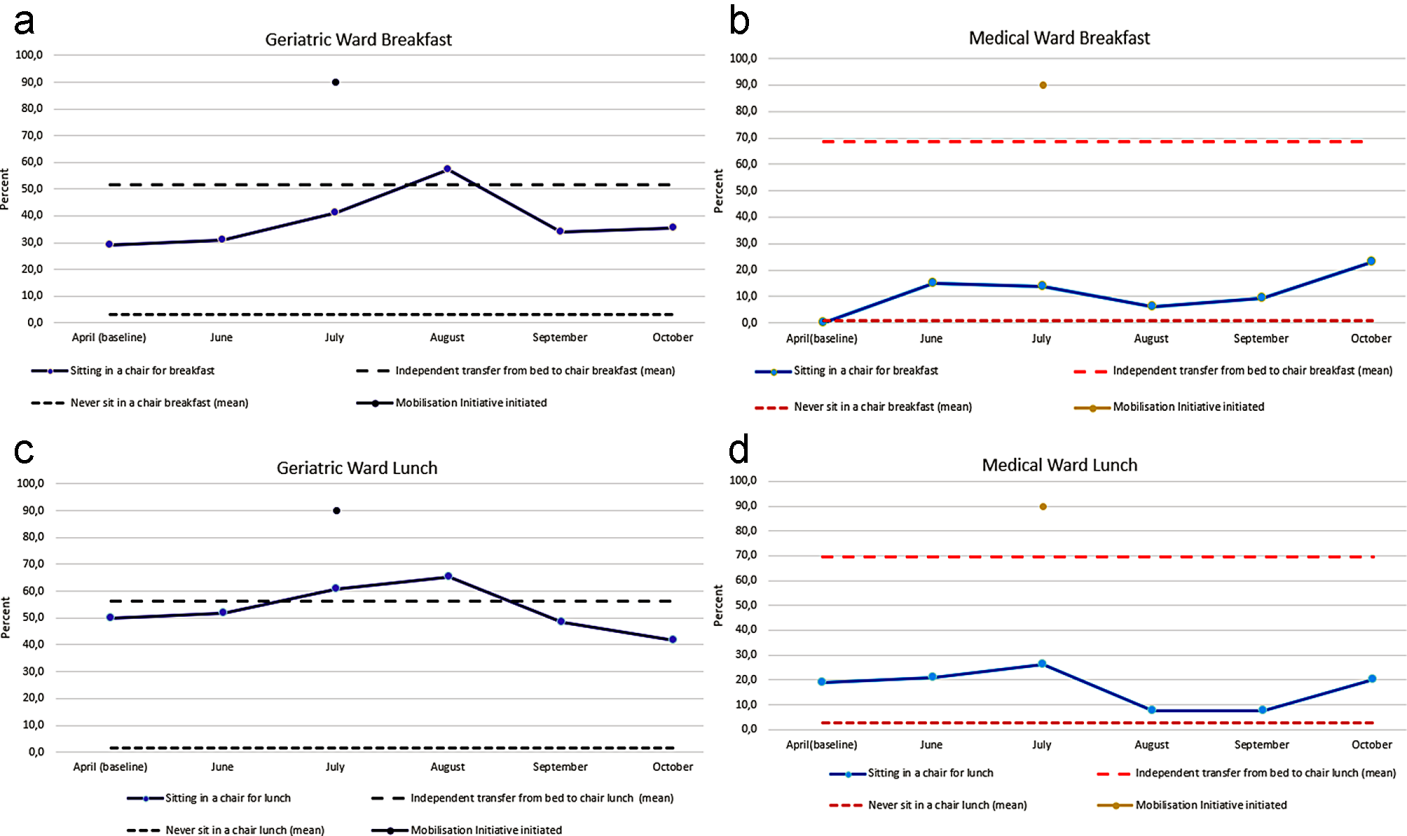
**a** Mobilisation level of patients at the geriatric ward for breakfast. **b** Mobilisation level of patients at the medical ward for breakfast. **c** Mobilization level of patients at the geriatric ward for lunch. **d** Mobilisation level of patients at the medical ward for lunch

A mean of 51.6% (breakfast) and 56.3% (lunch) from the geriatric ward (Fig. 3a and c) and 68.7% and 69.5% from the medical (Fig. 3b and d) ward reported that they were able to independently transfer from the bed to a chair for breakfast and lunch, respectively. Less than 4.0% of the patients in the two wards declared that they never sat in a chair for breakfast and lunch, leaving more than 96.0% able to complete the transfer with supervision or with physical help from one or two persons (for details please see Appendix 5).

For the observed level of mobilisation at baseline, 29.0% in the geriatric ward and 0.0% in the medical ward consumed their breakfast while sitting in a chair. During the study period, the highest percentage of patients who consumed their breakfast sitting in a chair was 57.1% in the geriatric ward (Fig. 3a) and 23.1% in the medical ward (Fig. 3b). Both occurred after the initiation of the Mobilisation Initiative in July.

At baseline, the percentage of patients sitting in a chair for lunch was 50.0% in the geriatric ward (Fig. 3c) and 18.8% in the medical ward (Fig. 3d). The highest percentage of patients sitting in a chair for lunch during the study period was 65.4% at the geriatric ward (Fig. 3c) and 26.3% at the medical ward (Fig. 3d), respectively. Both after the initiation of the Mobilisation Initiative in July.

At baseline, a mean of 89.4% of the patients at the geriatric ward had a chair in their hospital room, compared to a mean of 74.4% of the patients at the medical ward. According to data from the entire monitoring period from baseline in April to the end of the period in October, an average of 90.7% of the patients in the geriatric ward and 68.2% in the medical ward had a chair in their hospital room. Data on chair placement were not available at baseline. From June to October, an average of 61.4% of the chairs in the geriatric ward and 26.3% in the medical ward were placed bedside (for details please see Appendix 6).

The number of chairs in the room at lunch was higher than at breakfast, with an average of 92.5% compared to 89.3% at the geriatric ward. Similarly, the number of chairs in the room was, on average, 71.7% at lunch and 65.5% at breakfast in the medical ward.

#### Patients’ reflections of consuming their meal while sitting in a hospital bed

During the analysis of the patients’ reflections on the reason for consuming their meal while sitting in a hospital bed, a wide range of barriers were identified, and three main themes emerged: “The experience of no alternative,” “The lack of help transferring to and from the chair,” and “The Covid-19 pandemic’s influence on mobilisation.”

##### Theme I: "The experience of no alternative"

This theme emerged from the context of the asymmetric patient–caregiver relationship. Simply put, patients rarely questioned why their meals were served to be consumed in bed. The patients only rarely reflected on this matter before being prompted to reflect. They simply just accepted the situation as context-related or they unintendedly interpreted the serving of the meal as related to their treatment. Statements such as *“I don’t know*,* I was never offered anything else”* and *“No one asked me to sit in a chair*,* and apparently*,* they are satisfied with me sitting here in bed”* were often the initial reflections.

Additionally, patients expressed hesitation in getting out of bed because they did not know when it would be possible to get back for a rest, and they experienced no alternative to staying in bed: *“They asked me where I wanted to eat: In bed or in the chair. I said in bed because if I get in the chair I won’t get back until after 5 hours”* and *“I guess no one had the time to lift me over there.”*

Furthermore, simply not having a chair within sight resulted in acceptance from the participants of staying in bed. *“I had no chair to sit in”* and *“We only have one chair in this room*.” Hence, the practical design of the hospital room and the absence of chairs lead to situated acceptance without further reflection. *“I don’t know*,* the meal was served here”* and *“I had it served here. I usually sit in a chair and eat and usually I also cook it myself”* are examples of situated acceptance.

##### Theme 2: “The lack of help transferring to and from the chair”

The patients’ unmet need for help transferring provides a basis for this theme. Although the patients have the motivation and the opportunities for help are present, such as the Mobilisation Initiative, the patients are not offered the help needed. Patient statements confirm this: *“I’m dizzy and must be careful when I walk*,*” “I can’t. My strength doesn’t allow it and I am very unsteady on my feet*,*”* and *“I don’t know. There was no one to help me over there and I can’t do it myself.”* Consequently, patients were bound to their bed for mealtimes, and they accepted the situation without further questioning.

##### Theme 3: “The Covid-19 pandemic’s influence on mobilisation”

This theme emerges from the consequences of the closed dayrooms in the wards due to the Covid-19 pandemic, which results in patients not having an alternative to consuming their meal in their hospital room. The hospital room, with limited space and close to other patients and their personal needs, did not encourage getting out of one’s own private space in bed, as expressed by the patients: *“It’s a small room. Other patients must be washed and if I had to sit in a chair*,* I would have to sit and watch him being washed*,*” “I like sitting here. I’ve gotten used to that. There was a week when there was a terrible commotion and then I sat down here*,*”* and *“You can’t sit in a chair here. It is unhygienic and there is nothing to look at. There ought to be a canteen where you can look outside*,* sit and talk to other people.”*

The lack of access to the dayroom also limited the general mobilisation of the patients, decreasing their activity levels during hospitalisation. The patients immediately accepted this premise, and neither they nor the nursing staff sought alternatives to the missing mobilisation: *“There is Corona. I usually sit in the living room. Previously*,* I used to pick up my food myself*,*” “I was not offered that. I asked if there wasn’t a living room I could sit in*,* I was told there wasn’t*,*”* and *“I don’t know. I sat very well here. I would rather sit in the living room*,* which is cosier.”*

### Focus group interviews, facilitators, and barriers

The staff expressed three facilitators for the patient mobility: interdisciplinary collaboration, if the patients felt a purpose of being mobilised, and patient motivation in seeing other patients mobilised. The latter was verbalised by the nursing staff, the occupational therapist, and the physiotherapist during the focus group interviews: *“What I really like is when a cognitively impaired patient is sparring with another patient sitting across from her*,* like: ‘This is how you do it’*,*” “They mirror each other*,*”* and *“They do what the others do.* Examples of interdisciplinary collaboration were expressed by the nursing staff and the physiotherapist: *“If we have days where we have fewer staff in the nursing team and can’t assist each other*,* we also engage physiotherapists for mobilisation” … “an example was with a heavy patient where I called for the physiotherapists. None of us knew what she (the patient) was capable of”*,* “We (the physiotherapist) had tried earlier but she (the patient) was very anxious with a lot of pain*,* so it was difficult to get her up” … “she had now reached a point where she felt confident with us (the physiotherapists) too*,* and we managed to get her up in collaboration”.*

Barriers to mobilisation of the patients were described as lack of time among the nursing staff, reduced number of nursing staff at work, lack of walking aids, and lack of chairs. The lack of time was expressed by a nursing staff: *“After all*,* breakfast must be served from 8-9.30 and we don’t have time to serve breakfast longer than that…it is physically impossible to manage to get everyone ready for breakfast. That’s why you have to say: ‘ok*,* I will change your diaper and then you will have to sit in your bed for breakfast because I have to prioritise others’.”*

Examples of solutions proposed to increase mobilisation were regular interdisciplinary meetings with nursing staff and physiotherapists with the focus of the patients’ mobilisation level, welcoming the patients at admission with briefing of the understanding that everyone sits in a chair at mealtimes, and postponement of morning toilette until after breakfast.

### Formal education, awareness of mobilisation survey, and physiotherapists’ evaluation of the Mobilisation Initiative

Less than 1/3 of the nursing staff at the two wards attended the formal education organised on two different days. In the following discussion, none expressed a lack of competencies or confidence with mobilisation. Lack of time and lack of nursing staff were the main barriers expressed by the nursing staff, and interprofessional collaboration was the main facilitator.

In June, the surveys of awareness of mobilisation were completed by 10 (34.5%) of the nursing staff at the geriatric ward and 7 (23.3%) of the nursing staff at the medical ward. The surveys in December were completed by 8 (24.2%) of the nursing staff at the geriatric ward and 8 (26.7%) at the medical ward. Most of the participants who completed the survey were employed within the last two years (geriatric ward: 52.9%, medical ward: 71.4%), and according to the information on working years at the wards, all but one of the participants was not the same in June and December.

Evaluations of the Mobilisation Initiative were conducted with the physiotherapists at regular intervals about every 5 weeks. The evaluations highlighted barriers to mobilisation, such as the lack of chairs present at the bedside and the nursing staff’s approach to the patients’ level of mobilisation at mealtimes. The latter was expressed by the physiotherapist: *“The patients had their meal served in bed without being asked or helped to sit in a chair.”*

### The Mobilisation Initiative

The Mobilisation Initiative was launched in July with physiotherapists assisting nursing staff in mobilising patients to sit in a chair for breakfast and lunch as a supplement to usual practice. In the period of July–December, the Mobilisation Initiative at the geriatric ward had, on average, 2.02 (SD 0.91) referrals every morning, and an average of 1.84 (SD 0.63) patients were mobilised. At the medical ward, an average of 0.65 (SD 0.30) patients were referred in the morning, and 0.69 (SD 0.31), on average, were mobilised, as some patients were mobilised without referral from the nursing staff. The months with the highest number of patients mobilised through the Mobilisation Initiative (average per morning) were August (2.32 patients) and September (2.59 patients) at the geriatric ward and September (0.91 patients) and October (0.90 patients) at the medical ward. The Mobilisation Initiative was only used sparsely for lunch, with 0.21 (SD 0.22) patients mobilised on average at the geriatric ward and 0.02 (SD 0.06) patients at the medical ward. In October and November, the geriatric ward referred no patients to the Mobilisation Initiative at lunch. The medical ward discontinued the use of the Initiative at lunch in August, with no referrals in September, October, November, and December. The Mobilisation Initiative for breakfast continued throughout the study period at both wards. No serious or non-serious adverse events or unintended effects were observed in relation to the Mobilisation Initiative.

In the Mobilisation Initiative, the barriers were that patients were not ready for mobilisation due to morning routines, the nursing staff did not have an overview of the need for help and therefore did not ask for help mobilising the patients, and the patients had their meal served in bed without being asked or helped to sit in a chair and without involving the Mobilisation Initiative.

## Discussion

This study showed low out-of-bed mobilisation rates for mealtimes, even though more than 50% of the patients reported that they were able to independently transfer from the bed to a chair, and 96.0% reported that they could complete the transfer with supervision or with physical help from one or two persons. Moreover, we identified possible barriers to mobilisation of patients at mealtimes in the form of the absence of bedside chairs and lack of interprofessional collaboration. Finally, although a Mobilisation Initiative was initiated to support the nursing staff at the wards with mobilisation at mealtimes, the initiative did not even lead to mobilisation of those who reported being able to transfer independently from the bed to a chair.

The low mobilisation rate could partly be related to an environmental barrier, as not all patients had chairs in their hospital rooms. Although more than 96.0% of the patients were able to sit in a chair at mealtimes with or without transfer support, only 90.7% of the patients at the geriatric ward and 68.2% at the medical ward had a chair in their hospital room, and far from all of these were placed bedside or near the table where the meal was served. This is in line with former studies describing the lack of chairs as a barrier to mobilisation [29, 30]. However, the lack of chairs cannot be interpreted as the only reason for the low mobilisation rate of patients. Therefore, in line with former findings, factors such as cultural, physical, organisational, and other environmental barriers seem to be important factors affecting the lack of mobilisation of patients in the present study [24–26, 29, 30].

Multiple findings in the present study underlined that the culture in the wards was a barrier to mobilisation. The patients experienced being given no alternative to having their meal served in bed, which was supported by observations in the wards. Simply put, the nursing staff accepted that the patients consumed their meal in bed and that it was not culturally embedded that patients were encouraged to get out of bed. This resembles findings in prior studies identifying encouragement and support from others as important facilitators for mobilisation and likewise as important barriers to mobilisation when absent [24, 30, 31]. Cultural aspects are difficult to change, and this could explain the limited use of the Mobilisation Initiative [32].

The patients in the geriatric ward were older and less independent than the patients in the medical ward. However, more patients were mobilised to a chair during mealtimes in the geriatric than in the medical ward. This is counterintuitive but might be explained by geriatrics as a specialty being characterized by its focus on rehabilitation and physical function [33]. Additionally, the geriatric specialty involves the collaboration of a multidisciplinary team, including physiotherapists, occupational therapists, nurses, and doctors, working together on the patient’s treatment, rehabilitation, and long-term care plan [33]. Therefore, physiotherapy is a part of the in-hospital treatment in the geriatric ward without the need for referrals. Moreover, the Mobilisation Initiative was used more frequently by the geriatric ward, despite both wards having the same access to and promotion of the initiative. A reason for the limited use of the Mobilisation Initiative at the medical ward could be that most of the patients did not need help transferring to a chair or only needed supervision, which possibly left the nursing staff with less focus on encouraging these patients to mobilise. However, as a non-negligible portion of the patients were still not mobilised for mealtimes, this underlines the missing focus on mobilisation and/or prioritisation of mobilisation and may suggest that no profession undertook the responsibility for mobilising the patients, as suggested in a previous study [25].

The lack of mobilisation of otherwise independent patients has also been described in previous studies [5, 16–20]. In addition, our findings that one reason for lack of mobilisation is lack of support from staff is well in line with prior findings that underline the importance of support from staff [25, 31]. It is clear from our study that there is a lack of awareness of older adults’ needs regarding mobilisation. On the one hand, our interviews underlined that older adults were afraid of mobilising on their own, and on the other hand, the health care staff did not make use of the Mobilisation Initiative.

The Mobilisation Initiative was developed and tested as an add-on to usual practice in the present study, as interprofessional collaboration and shared responsibility have previously been emphasised as one of the main determinants for increasing mobilisation [25], speaking in favour of the chosen method in this study. Keeping the barriers of mobilisation in mind, including the cultural and organisational barriers, choosing a different approach with physiotherapists mobilising all patients without the need for referrals and collaboration of the nursing staff, would most likely have increased the number of patients mobilised. However, the consequence of such an approach would be that mobilisation of the patients would only be a physiotherapist’s responsibility. Physiotherapists are not present in the wards at all hours. Consequently, this could leave the patients less mobile during hours, with no physiotherapists present. In contrast, seeing more patients out of bed could have the positive effect of increased awareness of mobilisation among the staff and patients, which was also expressed as a facilitator by the nursing staff in the focus group interviews.

The attendance at the formal education was lower than anticipated. Thus it did not fulfil its purpose of enhancing confidence, competencies, and awareness of mobilisation among the nursing staff. It is possible that formal education was not the optimal approach for this purpose. Accordingly, a higher degree of attendance at the formal education sessions could have encouraged more nursing staff to complete the surveys and to change their behaviour towards more focus on mobilisation. Involvement of the nursing staff in planning the format of the intervention could have been beneficial and might have changed the nursing staff’s sense of ownership of the project. A minor increase in mobilised patients was observed during the Mobilisation Initiative, but the tendency did not last, even though the nursing staff knew they were part of a project and mobilisation was registered. It is possible that after a long period of the Covid-19 pandemic, the increased workload, and a lack of staff could had devaluated mobilisation as a core task among the nursing staff, who might have considered it a physiotherapeutic task. This would be in line with previous findings that no health care profession considers mobilisation their core task [25, 34]. This underlines the need for managerial support in prioritising mobilisation as a collective practice with a mutual understanding of the importance of, and thereby a culture of, mobilisation as a shared responsibility [25]. Managerial support is essential to ensure the mobilisation of patients at mealtimes and during hospitalisation in general. Managerial support will increase the possibility of maintaining a continuous focus on mobilisation and thereby a greater opportunity to proceed with the Mobilisation Initiative and new initiatives. The latter could be in the form of information materials for patients and relatives about the importance of mobilisation, involving volunteers and relatives to support mobilisation during hospitalisation, and not least, involving the nursing staff in the planning of interventions to increase focus on mobilisation for both staff and patients. Thus, to ensure sustainability, focus must be put on ensuring that mobilisation is regarded both as being important and a shared responsibility.

### Strengths and limitations

A strength of this study is that the mobilisation of patients at mealtimes was assessed through both observation and patient interviews for consecutive months. However, it is not known whether the two monthly days of observation and interviews are representative of the rest of the days of hospitalisation. The present study was performed at a single centre; therefore, the organisational and local procedures could potentially influence the results. Hence, generalisation of the results should be performed with caution. However, the overall results of a missed potential to mobilise patients during mealtimes were obtained from two separate wards. This indicates that the missed potential could be interpreted as a generic potential.

Patients with delirium and patients who were isolated were excluded from participation. This is a limitation of the study, as they are periodically part of the group of patients in both the geriatric and the medical ward to a greater or lesser extent. These patients may be mobilised less than those who are not delirious or isolated. Hence, our results might underestimate the potential for mobilisation. In alignment with this, in a study in older adults acutely admitted for medical illness Fisher et al. [35] found delirium to be associated with less steps during hospitalisation.

Because of the denied access to the separate dayroom with dining tables and chairs due to the Covid-19 restrictions, the patients were restricted to consuming their meals in their hospital room, with limited space for both staff and patients. Thus, this was unlike usual practice in the wards, and the results of this study are possibly negatively influenced by this factor.

Furthermore, the surveys proved not to be the ideal method for gathering information on the staff’s sense of competencies and confidence in mobilisation on an individual level. This might be due to a sense of risk of exposing one’s own lack of competencies or confidence, or due to busyness. The individual’s sense of competencies and confidence in mobilisation within the nursing staff proved impossible to measure. The low participation rate and the lack of continuity of staff completing the surveys meant that the results could not serve as representative of the entire nursing staff or give a reliable picture of the development of awareness of mobilisation among the nursing staff.

## Conclusion

This study adds new knowledge regarding the lack of in-hospital mobilisation of older patients admitted to geriatric and medical departments. Most patients reported that they could independently transfer from the bed to a chair. Nevertheless, only a minority of the patients consumed their meals outside the bed, even though mealtimes were an obvious opportunity for the patients to be mobilised, either independently or with the assistance of health care professionals. A potential for a Mobilisation Initiative is present. However, it must be interdisciplinarily and organisationally anchored and prioritised for further investigation of its effectiveness.

### Electronic supplementary material

Below is the link to the electronic supplementary material.

Supplementary Material 1

## Data Availability

The datasets used and/or analysed during the current study are available from the corresponding author on reasonable request.

## Abbreviations

HADs Hospital: Associated Disabilities
N: Numbers
PI: Primary Investigator
REDCap: Research Electronic Data Capture System
SD: Standard Deviations
