# Supplementary material for: Hospital-induced immobility – a backstage story of lack of chairs, time, and assistance
Source: BMC Geriatr. 2024 Aug 24;24:704. doi: 10.1186/s12877-024-05286-6 (PMC11344450; doi:10.1186/s12877-024-05286-6)
Supplement: Supplementary file 1 — Supplementary Material 1 [file 12877_2024_5286_MOESM1_ESM.docx]

# **Appendix 1: Self-reported Level of Patient Mobilisation**

| Record ID |  |
| --- | --- |
| Date |  |
| Ward | □ Geriatric |
|  | □ Medical |
| Age |  |
| Day of hospitalisation |  |
| Is the patient known to have cognitive problems? | □ Yes |
|  | □ No |
| Is there a chair present in the room for the patient? | □ Yes |
|  | □ No |
| Is the chair placed bedside? | □ Yes |
|  | □ No |
| How did you have your breakfast/lunch served? | □ Lying in bed |
|  | □ Sitting in bed |
|  | □ Sitting on the edge of the bed |
|  | □ Sitting in a chair |
|  | □ Did not have breakfast/lunch |
| What was the reason you did not have breakfast/lunch? |  |
| Where did you consume your breakfast/lunch | □ Lying in bed |
|  | □ Sitting in bed |
|  | □ Sitting on the edge of the bed |
|  | □ Sitting in a chair |
|  | □ Did not have breakfast/lunch |
| What was the reason you did not sit in a chair for breakfast/lunch? |  |
| How do you get from the bed to a chair? | □ Independently |
|  | □ With supervision |
|  | □ With the physical help of one person |
|  | □ With the physical help of two persons |
|  | □ Never sit in a chair |
| Do you need any aids when getting from the bed to a chair? | □ Yes |
|  | □ No |
|  | □ I don’t know |

# **Appendix 2: Observation Check List**

| **Date:** | Hospital room and bed:  Ward:   - Geriatric - Medical |
| --- | --- |
| **Meal** | **How the meal was consumed** |
| **Breakfast** | - Sitting in bed - Sitting on the edge of the bed - Sitting in a chair - Did not have breakfast |
| **Lunch** | - Sitting in bed - Sitting on the edge of the bed - Sitting in a chair - Did not have lunch |

# **Appendix 3: Interview Guide for Focus Group Interviews**

**Aim**

- To clarify facilitators and barriers for mobilisation of geriatric and medical inpatients
- To find proposed solutions for optimising mobilisation of geriatric and medical inpatients

**Participants**

Two care assistants, one nurse, one occupational therapist, and one physiotherapist from the geriatric and the medical ward, respectively.

Interview moderator: Dorte Barfred Stisen (DBS)

Observer: Katrine Storm Piper (KSP)

**Interview Guide**

| **Topic** | **Content** | **Time** |
| --- | --- | --- |
| **Presentation** | - Presentation of the project: KSP - Presentation of the focus group interview: DBS | 5 min |
| **Mobilisation of geriatric and medical inpatients in general** | - Tell us about how you experience mobilisation of the patients at your ward - Tell us about what works in relation to mobilisation of the patients - Tell us about what does not work in relation to mobilisation of the patients - How is mobilisation prioritised in your daily routine? - Who has the responsibility for mobilisation of the patients? | 10 min |
| **Data from surveys on patients’ self-reported level of mobilisation (quantitative)**  **Selected data presented illustratively, e.g. as pie charts** | - Comments on the results? - Are you surprised by the results?   - If not, why?   - If yes, in which way/which areas? | 10 min |
| **Data from surveys on patients’ self-reported level of mobilisation (qualitative) (patient statements)**  **Selected patient statements presented through printouts** | - Comments on the statements? - Are you surprised by the statements?   - If not, why?   - If yes, in which way/which areas? - Discussion of quantitative and qualitative data | 15 min |
| **Proposed solutions** | - Which solutions could be proposed to increase mobilisation?   - In the short term (what can be changed now without a larger intervention?)   - In the long term (what would you like to change in the future e.g. with a larger intervention?) - If the Department of Occupational Therapy and Physiotherapy in any way could help with some of the barriers for mobilisation, how do you think this could come about? | 15 min |
| **Rounding-off** | - How was it to participate in this interview? | 5 min |

# **Appendix 4: Survey on Awareness of Mobilisation**

| Record ID |  |
| --- | --- |
| Date |  |
| Which ward do you work in? | □ Geriatric |
|  | □ Medical |
| When were you hired at the ward? | ____________________________  (Write date and year for the first day of work, also if you are a student) |
| What is your profession? | □ Nurse |
|  | □ Care assistant |
|  | □ Student nurse |
|  | □ Student care assistant |
|  | □ Other |
| If other, describe |  |
| Do you feel confident mobilising your patients to sit in a chair at mealtimes? | □ Never |
|  | □ Less than half of the time |
|  | □ Half of the time |
|  | □ More than half of the time |
|  | □ Every time |
| To what degree do you feel prepared to do a safe transfer from the bed to a chair with a patient you do not know? | □ To a very low degree |
|  | □ To a low degree |
|  | □ Neither/nor |
|  | □ To a high degree |
|  | □ To a very high degree |
| How often do you talk to your colleagues about mobilisation?” | □ Never |
|  | □ Less than half of the time |
|  | □ Half of the time |
|  | □ More than half of the time |
|  | □ Every time |
| Which initiatives does your department employ to maintain a focus on mobilisation? |  |

# **Appendix 5: Key observational variables**

| Breakfast  Medical ward | | | | | | |
| --- | --- | --- | --- | --- | --- | --- |
|  | **April (baseline)** | **June** | **July** | **August** | **September** | **October** |
| Chairs, n (%) | n=39 | n=21 | n=24 | n=28 | n=15 | n=15 |
| Chairs present | 29 (74.4) | 13 (61.9) | 18 (75.0) | 18 (64.3) | 7 (46.7) | 8 (53.3) |
| Chairs bedside | NA | 2 (15.4) | 5 (27.8) | 4 (22.2) | 1 (14.3) | 4 (50.0) |
| Consuming breakfast, n (%) | n=30 | n=27 | n=29 | n=32 | n=21 | n=13 |
| Lying in bed | 0 (0) | 3 (11) | 0 (0) | 0 (0) | 1 (4.8) | 0 (0) |
| Sitting in bed | 10 (33.3) | 4 (14.8) | 8 (27.6) | 5 (15.6) | 9 (42.9) | 2 (15.4) |
| Sitting on the edge of the bed | 20 (66.7) | 16 (59.3) | 17 (58.6) | 25 (78.1) | 9 (42.9) | 8 (61.5) |
| Sitting in a chair, n (%) | 0 (0) | 4 (14.8) | 4 (13.8) | 2 (6.3) | 2 (9.5) | 3 (23.1) |
| Do not know | 0 (0) | 0 (0) | 0 (0) | 0 (0) | 0 (0) | 0 (0) |
| Transfer from bed to chair, n (%) | n=33 | n=21 | n=24 | n=27 | n=14 | n=15 |
| Independent | 23 (69.7) | 16 (76.2) | 16 (66.7) | 19 (70.4) | 10 (71.4) | 8 (53.3) |
| With supervision | 0 (0) | 1 (4.8) | 0 (0) | 1 (3.7) | 0 (0) | 0 (0) |
| With the physical help of one person | 6 (18.2) | 3 (14.3) | 5 (20.8) | 5 (18.5) | 2 (14.3) | 5 (33.3) |
| With the physical help of two persons | 2 (6.1) | 0 (0) | 3 (12.5) | 2 (7.4) | 1 (7.1) | 1 (6.7) |
| Never sit in a chair | 0 (0) | 1 (4.8) | 0 (0) | 0 (0) | 0 (0) | 0 (0) |
| Do not know | 2 (6.1) | 0 (0) | 0 (0) | 0 (0) | 1 (7.1) | 1 (6.7) |

| Breakfast  Geriatric ward | | | | | | |
| --- | --- | --- | --- | --- | --- | --- |
|  | **April (baseline)** | **June** | **July** | **August** | **September** | **October** |
| Chairs, n (%) | n=47 | n=23 | n=29 | n=24 | n=39 | n=15 |
| Chairs present | 42 (89.4) | 17 (73.9) | 27 (93.1) | 23 (95.8) | 36 (92.3) | 13 (86.7) |
| Chairs bedside | NA | 11 (64.7) | 14 (51.9) | 17 (73.9) | 19 (52.8) | 7 (53.8) |
| Consuming breakfast, n (%) | n=31 | n=26 | n=34 | n=28 | n=41 | n=17 |
| Lying in bed | 0 (0) | 1 (3.8) | 3 (8.8) | 1 (3.6) | 2 (4.9) | 2 (11.8) |
| Sitting in bed | 9 (29.0) | 5 (19.2) | 4 (11.8) | 2 (7.1) | 8 (19.5) | 3 (17.6) |
| Sitting on the edge of the bed | 13 (41.9) | 12 (46.2) | 12 (35.3) | 9 (32.1) | 16 (39.0) | 6 (35.3) |
| Sitting in a chair | 9 (29.0) | 8 (30.8) | 14 (41.2) | 16 (57.1) | 14 (34.1) | 6 (35.3) |
| Do not know | 0 (0) | 0 (0) | 0 (0) | 0 (0) | 1 (2.4) | 0 (0) |
| Transfer from bed to chair, n (%) | n=31 | n=23 | n=28 | n=24 | n=38 | n=15 |
| Independent | 19 (61.3) | 10 (43.5) | 12 (42.9) | 13 (54.2) | 19 (50.0) | 9 (60.0) |
| With supervision | 0 (0) | 1 (4.3) | 3 (10.7) | 0 (0) | 3 (7.9) | 0 (0) |
| With the physical help of one person | 9 (29.0) | 8 (34.8) | 7 (25.0) | 8 (33.3) | 12 (31.6) | 3 (20.0) |
| With the physical help of two persons | 2 (6.5) | 3 (0) | 5 (17.9) | 1 (4.2) | 3 (7.9) | 2 (13.3) |
| Never sit in a chair | 0 (0) | 1 (4.3) | 1 (3.6) | 1 (4.2) | 1 (2.6) | 1 (6.7) |
| Do not know | 1 (3.2) | 0 (0) | 0 (0) | 1 (4.2) | 0 (0) | 0 (0) |

| Lunch  Medical ward | | | | | | |
| --- | --- | --- | --- | --- | --- | --- |
|  | **April (baseline)** | **June** | **July** | **August** | **September** | **October** |
| Chairs, n (%) | n=31 | n=16 | n=19 | n=23 | n=13 | n=10 |
| Chairs present | 27 (87.1) | 10 (62.5) | 15 (78.9) | 17 (70.8) | 8 (61.5) | 4 (40.0) |
| Chairs bedside | NA | 5 (50.0) | 4 (25.0) | 2 (11.8) | 2 (25.0) | 2 (50.0) |
| Consuming lunch, n (%) | n=17 | n=24 | n=19 | n=26 | n=13 | n=15 |
| Lying in bed | 0 (0.0) | 2 (8.3) | 0 (0.0) | 0 (0.0) | 0 (0.0) | 0 (0.0) |
| Sitting in bed | 2 (11.8) | 6 (25.0) | 7 (36.8) | 6 (23.1) | 6 (46.2) | 0 (0.0) |
| Sitting on the edge of the bed | 12 (70.6) | 11 (45.8) | 7 (36.8) | 18 (69.2) | 5 (38.5) | 8 (80.0) |
| Sitting in a chair | 3 (17.6) | 5 (20.8) | 5 (26.3) | 2 (7.7) | 1 (7.7) | 2 (20.0) |
| Do not know | 0 (0.0) | 0 (0.0) | 0 (0.0) | 0 (0.0) | 1 (7.7) | 0 (0.0) |
| Transfer from bed to chair, n (%) | n=24 | n=16 | n=20 | n=23 | n=13 | n=10 |
| Independent | 17 (70.8) | 12 (75.0) | 13 (65.0) | 17 (73.9) | 7 (53.8) | 8 (80.0) |
| With supervision | 1 (4.2) | 2 (12.5) | 0 (0.0) | 1 (4.3) | 0 (0.0) | 0 (0.0) |
| With the physical help of one person | 2 (8.3) | 1 (6.3) | 4 (20.0) | 3 (13.0) | 2 (15.4) | 1 (10.0) |
| With the physical help of two persons | 4 (14.8) | 0 (0.0) | 2 (10.0) | 2 (8.7) | 1 (7.7) | 1 (10.0) |
| Never sit in a chair | 0 (0.0) | 1 (6.3) | 1 (5.0) | 0 (0.0) | 1 (7.7) | 0 (0.0) |
| Do not know | 0 (0.0) | 0 (0.0) | 0 (0.0) | 0 (0.0) | 2 (15.4) | 0 (0.0) |

| Lunch  Geriatric ward | | | | | | |
| --- | --- | --- | --- | --- | --- | --- |
|  | **April (baseline)** | **June** | **July** | **August** | **September** | **October** |
| Chairs, n (%) | n=41 | n=18 | n=22 | n=21 | n=35 | n=10 |
| Chairs present | 38 (92.7) | 17 (94.4) | 21 (95.5) | 20 (95.2) | 32 (91.4) | 8 (80.0) |
| Chairs bedside |  | 12 (70.6) | 12 (57.1) | 18 (90.0) | 19 (59.4) | 3 (37.5) |
| Consuming lunch, n (%) | n=22 | n=29 | n=28 | n=26 | n=37 | n=12 |
| Lying in bed | 0 (0.0) | 0 (0.0) | 0 (0.0) | 0 (0.0) | 0 (0.0) | 0 (0.0) |
| Sitting in bed | 3 (13.6) | 7 (24.1) | 3 (10.7) | 3 (11.5) | 8 (21.6) | 1 (8.3) |
| Sitting on the edge of the bed | 7 (31.8) | 7 (24.1) | 8 (28.6) | 5 (19.2) | 11 (29.7) | 6 (50.0) |
| Sitting in a chair | 11 (50.0) | 15 (51.7) | 17 (60.7) | 17 (65.4) | 18 (48.6) | 5 (41.7) |
| Don’t know | 1 (4.5) | 0 (0.0) | 0 (0.0) | 1 (3.8) | 0 (0.0) | 0 (0.0) |
| Transfer from bed to chair, n (%) | n=22 | n=18 | n=22 | n=21 | n=33 | n=10 |
| Independent | 13 (59.1) | 9 (50.0) | 9 (40.9) | 10 (47.6) | 22 (66.7) | 8 (80.0) |
| With supervision | 1 (4.5) | 1 (5.6) | 1 (4.5) | 2 (9.5) | 1 (3.0) | 0 (0.0) |
| With the physical help of one person | 6 (27.3) | 4 (22.2) | 8 (36.4) | 8 (38.1) | 8 (24.2) | 1 (10.0) |
| With the physical help of two persons | 1 (4.5) | 3 (16.7) | 4 (18.2) | 0 (0.0) | 2 (6.1) | 1 (10.0) |
| Never sit in a chair | 0 (0.0) | 1 (5.6) | 0 (0.0) | 1 (4.8) | 0 (0.0) | 0 (0.0) |
| Don’t know | 1 (4.5) | 0 (0.0) | 0 (0.0) | 0 (0.0) | 0 (0.0) | 0 (0.0) |

**Appendix 6: Availability of chairs month by month**

| Chairs at the geriatric ward | | | | |
| --- | --- | --- | --- | --- |
|  | **Chairs present breakfast** | **Chairs bedside breakfast** | **Chairs present lunch** | **Chairs bedside lunch** |
| Month monitored, n (%)^a^ |  |  |  |  |
| April(baseline) | 42 (89.4) |  | 37 (92.7) |  |
| June | 17 (73.9) | 11 (64.7) | 17 (94.4) | 12 (70.6) |
| July | 27 (93.1) | 14 (51.9) | 22 (95.7) | 12 (60.0) |
| August | 23 (95.8) | 17 (73.9) | 20 (95.2) | 18 (90.0) |
| September | 36 (92.3) | 19 (52.8) | 32 (91.4) | 19 (59.4) |
| October | 13 (86.7) | 7 (53.8) | 8 (80.0) | 3 (37.5) |

^a^The number of chairs present and the percentage of these placed bedside

| Chairs at the medical ward | | | | |
| --- | --- | --- | --- | --- |
|  | **Chairs present breakfast** | **Chairs bedside breakfast** | **Chairs present lunch** | **Chairs bedside lunch** |
| Month monitored, n (%)^a^ |  |  |  |  |
| April(baseline) | 29 (74.4) |  | 27 (87.1) |  |
| June | 13 (61.9) | 2 (15.4) | 10 (62.5) | 5 (50.0) |
| July | 18 (75.0) | 5 (27.8) | 15 (78.9) | 4 (25.0) |
| August | 18 (64.3) | 4 (22.2) | 17 (70.8) | 2 (11.8) |
| September | 7 (46.7) | 1 (14.3) | 8 (61.5) | 2 (25.0) |
| October | 8 (53.3) | 4 (50.0) | 4 (40.0) | 2 (50.0) |

^a^The number of chairs present and the percentage of these placed bedside
